# Supplementary material for: FTO promotes skeletal muscle differentiation and regeneration by regulating m6A-modified c-Myc
Source: bioRxiv. 2025 Dec 30:2025.12.30.696954. Preprint. [Version 1] doi: 10.64898/2025.12.30.696954 (PMC12776283; doi:10.64898/2025.12.30.696954)

bioRxiv preprint doi: https://doi.org/10.64898/2025.12.30.696954; this version posted December 30, 2025. The copyright holder for this preprint (which was not certified by peer review) is the author/funder, who has granted bioRxiv a license to display the preprint in perpetuity. It is made available under aCC-BY 4.0 International license.

Dey_Supplemental Table 1

Treatments	MYOG-positive cell number			MHC-positive cell number		
	Total Cells (12 fields)	Cells per field		Total Cells (12 fields)	Cells per field	
		Mean	SD		Mean	SD
**EV**	467	38.91	5.68	395	32.91	4.42
**FTO Ovx**	1012	84.33	8.26	828	69.00	5.86

bioRxiv preprint doi: https://doi.org/10.64898/2025.12.30.696954; this version posted December 30, 2025. The copyright holder for this preprint (which was not certified by peer review) is the author/funder, who has granted bioRxiv a license to display the preprint in perpetuity. It is made available under aCC-BY 4.0 International license.

Dey_Supplemental Table 2

Treatments	MYOG-positive cell number			MHC-positive cell number		
	Total Cells (12 fields)	Cells per field		Total Cells (12 fields)	Cells per field	
		Mean	SD		Mean	SD
**gap-NC**	550	45.83	8.35	536	44.47	5.91
**gap-FTO**	286	23.83	5.07	250	20.83	3.15

bioRxiv preprint doi: https://doi.org/10.64898/2025.12.30.696954; this version posted December 30, 2025. The copyright holder for this preprint (which was not certified by peer review) is the author/funder, who has granted bioRxiv a license to display the preprint in perpetuity. It is made available under aCC-BY 4.0 International license.

Dey Supplemental Figure 1

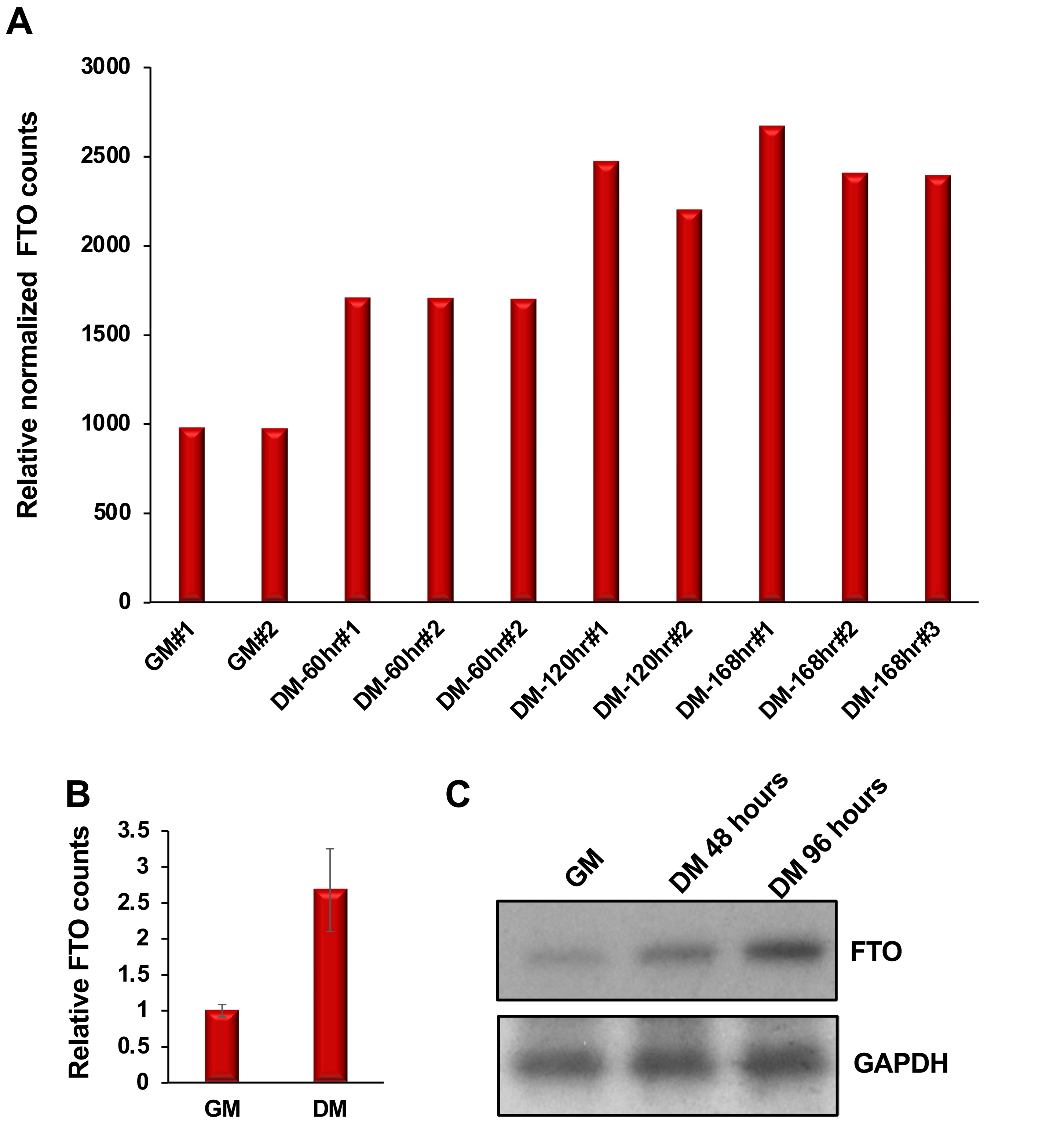

bioRxiv preprint doi: https://doi.org/10.64898/2025.12.30.696954; this version posted December 30, 2025. The copyright holder for this preprint (which was not certified by peer review) is the author/funder, who has granted bioRxiv a license to display the preprint in perpetuity. It is made available under aCC-BY 4.0 International license.

Dey_Supplemental Figure 2

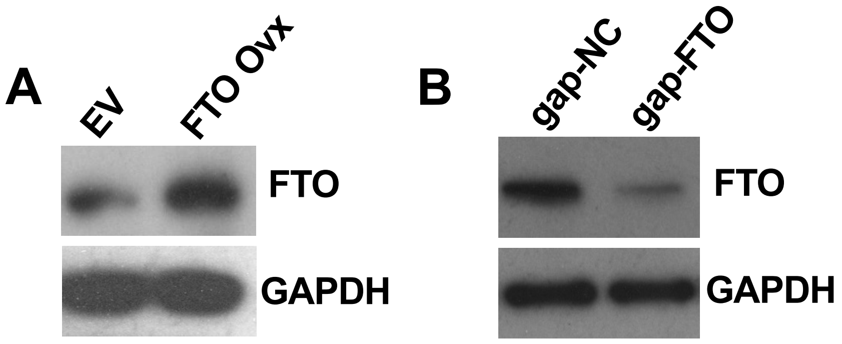

Supplement: Supplement 1 — Supplemental Figure 1: FTO is upregulated during C2C12 myoblast differentiation. (A) Normalized RNA-seq counts from undifferentiated C2C12 myoblasts (GM) and myoblasts differentiated in differentiation medium (DM) for 60, 120, and 168 hours are shown. (B) qRT-PCR analyses show that Fto is upregulated during C2C12 myoblast differentiation. Fto levels were first normalized to Gapdh, and fold changes were calculated relative to undifferentiated myoblasts (GM). DM indicates culture in differentiation medium for 96 hours. (C) FTO protein levels, as shown by Western blotting, also increase during mouse C2C12 myoblast differentiation. GAPDH served as a loading control. Supplemental Figure 2: (A) Western blotting showing that myoblasts transduced with a retroviral vector expressing FTO increase FTO protein level. (B) Western blot analysis showing that myoblasts treated with gap-FTO decrease FTO protein levels. Supplemental Table 1: Ectopic expression of FTO increases the number of MYOG- and MHC-positive cells. Supplemental Table 2: Knockdown of Fto decreases the number of MYOG- and MHC-positive cells. [file NIHPP2025.12.30.696954v1-supplement-1.pdf]
